# The relationship between stress, social capital and quality of education among medical residents

**DOI:** 10.1186/s13104-018-3387-5

**Published:** 2018-05-04

**Authors:** Charis Anastasiadis, Andreas Tsounis, Pavlos Sarafis

**Affiliations:** 1 “Konstantopoulio” General Hospital of Nea Ionia, Athens, Greece; 2 School of Psychology, The Aristotle University of Thessaloniki, Thessaloniki, Greece; 3 Department of Nursing, School of Health Sciences, Cyprus University of Technology, 15, Vragadinou Str, 3041 Limassol, Cyprus

**Keywords:** Hospital, Quality of education, Residents, Social capital, Stress

## Abstract

**Objective:**

The educational climate is a key factor in medical education. The study aims to examine the relationship between trainee doctors’ perceptions of hospital educational environment, stress and social capital. A cross-sectional study among 104 trainee doctors working in a Greek public hospital was conducted. According to the main hypotheses, perceptions of clinical training are positively associated with social capital and negatively with stress.

**Results:**

Perceptions of autonomy dimension of training quality was positively related to community participation, tolerance of diversity and total social capital. Perceptions of teaching and social support dimensions of the quality of education were positively correlated with community participation. All training quality subscales were negatively correlated with almost all working stress subscales. Analysis revealed significantly higher scores in autonomy perceptions for those who evaluated their undergraduate studies positively. Females had a significantly lower score in perceptions of teaching and social support scales.

## Introduction

The educational environment is as a key element for the quality of medical education [1]. The World Federation for Medical Education has mentioned as the most important components of the educational environment: the atmosphere, the learning opportunities and the availability of facilities [2]. Other researchers distinguish between physical environment (safety, shelter and other facilities), emotional climate (social support, absence of harassment) and intellectual climate (active participation, evidence-based learning) [3].

The transition to the hospital is stressful for most junior doctors. Long working hours, inadequate support from senior staff, new responsibilities, job insecurity and the fact that their mistakes are highly visible are among the most common stressors [4, 5].

Job and personal resources may buffer job demands that may lead to high-stress levels for junior doctors [5]. Job demands are these organizational factors that require constant physical and psychological effort. Job resources are the psychological, social and organizational aspects of work (e.g. autonomy, supervisory coaching). Personal recourses are those psychological and social elements that contribute to an individuals’ ability to control and impact on his/her environment successfully [6, 7]. Social capital (SC) is a resource on both individual and ecological level that may contribute to stress reduction and junior doctors’ adaptation to the educational environment.

SC is defined as “those features of social structures-such as interpersonal trust and norms of reciprocity and mutual aid—which act as resources for individuals and facilitate collective action” [8]. SC may help individuals’, groups’ and organizations’ function by enabling cooperative ventures and facilitating interpersonal interactions [9].

SC main theoretical approaches are the individualistic and the collective. According to the individualistic approach, people benefit from participating in social networks by gaining access to informational, emotional and verbal support [10]. In the collective approach, the emphasis is given to the group or to the community. Communities or organizations rich in social interactions have better economic, health and social indices [11].

The main distinction concerning its’ types is made between cognitive and structural SC [12]. Cognitive refers to perceptions, shared values, beliefs, and attitudes like trust, reciprocity, and tolerance. Structural SC refers to the way that people act in their social environment (e.g. level of participation, number of networks) [13]. Another distinction, based in the type of the connections, is between bonding (informal networks of strong ties, like family), bringing (informal networks of weaker ties like colleagues) and linking (formal networks) SC [14]. In both distinctions, the capacity of the individuals acting together and the potential of this cooperation is evaluated [15].

SC has beneficial effects for both individuals and organizations. Literature suggests an inverse relationship between higher SC and mental disorders [16]. Additionally, it is positively associated with educational attainment [17] and achievement [18]. In organizational studies, SC is positively related with job satisfaction [9, 19] and negatively with emotional exhaustion and turnover intention [20].

The theoretical model of the study is based on Job Demands-Resources theory according to which personal resources may buffer stress provoked from job demands [7]. The current study investigates the relation between trainee doctors’ perceptions of the hospital educational environment, stress and their SC. SC was treated as an ecological resource which was measured on individual level. Both its’ cognitive (e.g. feelings of safety) and structural (e.g. family, friends and work connections) elements were evaluated. According to our main hypotheses, residents’ positive perceptions of the educational environment are negatively associated with stress and positively with higher SC levels.

## Main text

### Methods

#### Participants and procedure

The study was conducted in ‘‘Konstantopoulio’’ General Hospital in Athens. In total, 149 anonymous questionnaires were distributed to all trainee doctors. The final sample consisted of 104 participants (Response Rate: 69.8%). The participants were informed on the purpose of the study and on confidentiality and they were free to participate, refuse or withdraw at any time.

#### Measures

A self-administrated questionnaire was used. At the first part, it contained questions recording socio-demographics and information concerning training, specialty, years of internship in the specific position and in total and satisfaction concerning internship.

Residents undergraduate education was evaluated with three questions ranked on a 6-point scale.

Residents’ SC was assessed by the Greek, validated, version of Social Capital Questionnaire (SCQ-G) [21]. SCQ-G is a 36 item ranked on a 4-point scale, with six subscales: (i) Local community participation; (ii) Feelings of safety; (iii) Family/friends connections; (iv) Value of life and social agency; (v) Tolerance of diversity; and (vi) Work connection. Higher scores indicate more SC.

Perceptions about the educational environment were assessed by the Greek, validated, version of Postgraduate Hospital Educational Environment Measure (PHEEM-G) [22]. PHEEM-G measures three domains of the clinical learning environment: (i) Perceptions of autonomy; (ii) Perceptions of teaching; (iii) and Perceptions of social support. It consists of 40 questions, ranked on a 5-point scale. Two opened type questions, concerning the satisfaction with the internship’s position and the expectations’ implementation were added during its’ validation process [22].

Stress was measured with Sources of Stress Scale (SSS) [23]. It consists of a total of 60 items measuring five dimensions: (i) Uncontrollable work situation; (ii) The organization of work and working conditions; (iii) Work-family conflict; (iv) Unfavorable relationships with colleagues; and (v) Interaction with patients. Each item ranked on a 3-point scale measuring the frequency and a 4-point scale measuring the tension of specific sources of stress.

#### Statistical analysis

Pearson’s coefficients were used exploring association of two continuous variables. Multiple linear regression analysis was used to dependent the training quality subscales. The regression equation included terms for sample characteristics as well as for SC and stress scales. Adjusted regression coefficients (β) with standard errors (SE) were computed. A stepwise method was used. All the reported p values were two-tailed. Analyses were conducted using SPSS (version 19.0).

## Results

The sample consisted of 104 medical interns with mean age 34.4 years old. Most of the participants were males (57.7%). Mean internship duration at the current site was 2.4 years and in general was 3.3 years. All the participants attended a full time residency in their current position. Concerning specialty, 23.1% were trained as pathologists, 15.4% as surgeons, 13.5% as general practitioners, 13.5% as radiologists, 6.7% as cardiologists, an equal percentage of 5.8% as obstetricians, urologists and orthopaedics, while 10.7% attended other specialties. Most of them (64.4%) have studied in Greece. More than half of the participants (51.9%) were satisfied with their current internship. Also, 34.6% of the participants agreed with the statement “My expectations I had when I started Medical school are fulfilled”, 55.8% with the statement “Medical school qualified me for dealing with the internship demands”, 39.4% with the statement “Starting my internship, I was already capable of dealing with daily clinical skills” and 50.0% with the statement “My undergraduate studies helped me in having better communication with my patients”.

Participants’ scores in all scales are presented in Table 1. Mean total SC score was 2.27. For the stress-related factors, mean scores for the frequency subscales ranged from 0.95 to 1.31 and for the intensity subscales ranged from 1.36 to 1.74. As far as training quality is concerned, the mean score for perceptions of autonomy was 23.98, for perceptions of teaching it was 28.45 and for perceptions of social support it was 21.04.

**Table 1:** Study scales descriptive

	Mean (SD)	Cronbach’s α
Social capital		
Participation in the community	1.70 (0.30)	0.73
Value of life and social agency	2.63 (0.36)	0.76
Feelings of safety	2.41 (0.57)	0.72
Family and friend relationships	2.43 (0.44)	0.81
Tolerance to diversity	2.30 (0.56)	0.88
Work relationships	2.67 (0.53)	0.76
Total SC	2.27 (0.25)	0.78
Uncontrollable work situations		
Frequency	1.15 (0.27)	0.72
Intensity	1.74 (0.54)	0.87
The organization of work and working conditions		
Frequency	1.31 (0.27)	0.70
Intensity	1.66 (0.63)	0.90
Conflict of work and family roles		
Frequency	1.31 (0.30)	0.73
Intensity	1.54 (0.60)	0.85
Unfavorable relationships with colleagues		
Frequency	0.95 (0.29)	0.72
Intensity	1.40 (0.54)	0.80
Interaction with patients		
Frequency	1.22 (0.36)	0.72
Intensity	1.36 (0.57)	0.77
Training quality		
Perceptions of autonomy	23.98 (8.85)	0.83
Perceptions of teaching	28.45 (10.72)	0.90
Perceptions of social support	21.04 (5.89)	0.71

Perceptions of autonomy scale was positively correlated with “Community Participation”, “Tolerance to Diversity” subscales and total SC score (Table 2). Thus, higher values in the aforementioned scores are associated with greater autonomy perceptions. Also, perceptions of teaching scale was positively correlated with “Community Participation” and perceptions of social support scale was positively correlated with “Community Participation” and total SC score. Also, all three training quality subscales were negatively correlated with almost all working stress subscales (frequency and intensity), indicating that participants’ less working stress is associated with greater satisfaction from their training.

**Table 2:** Correlation coefficients between training quality scales and social capital and working stress scales

	Perceptions of autonomy	Perceptions of teaching	Perceptions of social support
Social capital			
Participation in the community	0.28**	0.26**	0.29**
Value of life and social agency	0.13	− 0.03	0.13
Feelings of safety	0.10	0.12	0.16
Family and friend relationships	0.13	0.16	0.16
Tolerance to diversity	0.22*	− 0.01	0.17
Work relationships	− 0.01	− 0.01	0.05
Total SC	0.24*	0.10	0.25*
Working stress			
Uncontrollable work situations			
Frequency	− 0.21*	− 0.08	− 0.18
Intensity	− 0.27**	− 0.30**	− 0.26**
The organization of work and working conditions			
Frequency	− 0.36***	− 0.34***	− 0.41***
Intensity	− 0.34***	− 0.37***	− 0.32**
Conflict of work and family roles			
Frequency	− 0.26**	− 0.23*	− 0.24*
Intensity	− 0.29**	− 0.36***	− 0.14
Unfavorable relationships with colleagues			
Frequency	− 0.35***	− 0.23*	− 0.30**
Intensity	− 0.43***	− 0.41***	− 0.29**
Interaction with patients			
Frequency	− 0.08	0.02	− 0.11
Intensity	− 0.17	− 0.28**	− 0.27**

*p < 0.050; **p < 0.010; ***p < 0.001

When multiple linear regression was applied, significantly higher scores in Perceptions of Autonomy were found in those participants who were satisfied with their current internship, believed that their undergraduate studies helped them to have better communication with patients, feel that Medical school qualified them for dealing with the internship demands and their expectations from Medical school were fulfilled (Table 3). Also, greater values in SC total score and lower values in frequency subscale of “The organization of work and working conditions” and in intensity subscale of “Unfavorable relationships with colleagues” are associated with higher scores in Perceptions of Autonomy scale.

**Table 3:** Multiple linear regression results with training quality subscales as dependent variables

	β^a^	SE^b^	P
Perceptions of autonomy			
In general, I am very satisfied with my internship in the current site			
Disagree	0.00^c^		
Agree	4.53	1.54	0.004
My undergraduate studies helped me in having better communication with my patients			
Disagree	0.00		
Agree	4.49	1.34	0.001
Med school qualified me for dealing with the demands of my internship			
Disagree	0.00		
Agree	3.91	1.41	0.007
My expectations I had when I started Med school are fulfilled.			
Disagree	0.00		
Agree	3.76	1.56	0.018
Total SC	8.42	3.39	0.007
The organization of work and working conditions (frequency)	− 11.64	2.92	< 0.001
Unfavorable relationships with colleagues (intensity)	− 4.66	1.27	< 0.001
Perceptions of teaching			
Sex			
Males	0.00^c^		
Females	− 5.83	1.38	< 0.001
In general, I am very satisfied with my internship in the current site			
Disagree	0.00		
Agree	8.46	1.57	< 0.001
My expectations I had when I started Med school are fulfilled			
Disagree	0.00		
Agree	4.42	1.56	0.006
Participation in the community	8.40	2.33	< 0.001
The organization of work and working conditions(frequency)	− 13.83	3.65	< 0.001
Unfavorable relationships with colleagues (intensity)	− 5.87	1.31	< 0.001
Perceptions of social support			
Sex			
Males	0.00^c^		
Females	− 2.44	1.01	0.017
In general, I am very satisfied with my internship in the current site.			
Disagree	0.00		
Agree	2.42	1.04	0.022
Total SC	6.02	1.96	0.003
The organization of work and working conditions (frequency)	− 7.35	1.87	< 0.001

^a^Regression coefficient

^b^Standard error

^c^Indicates reference category

Females had a significantly lower score in perceptions of teaching scale compared to males. Moreover, higher scores in perceptions of teaching were found in participants who were satisfied with their current internship and indicated that the expectations from Medical school were fulfilled. The greater score in “Community Participation” and lower values in frequency subscale of “The organization of work and working conditions” and in intensity subscale of “Unfavorable relationships with colleagues” were associated with higher perceptions of teaching scores.

Females had a significantly lower score in perceptions of social support compared to males. Moreover, higher scores in perceptions of social support were found in participants who were satisfied with their current internship. Greater values in SC total score and lower values in “The organization of work and working conditions” were associated with higher scores in perceptions of social support.

## Discussion

The study investigated the relationship between medical residents’ perceptions of hospital educational environment, stress and SC. The results revealed a negative view of one’s role concerning perceptions of autonomy, need of some retraining for teaching perceptions and a perception of a not pleasant place for social support climate, according to PHEEM cut-off scores [1, 22]. Moreover, 68% of the participants declared that their expectations from Medical school were not fulfilled. The above is consistent with previous findings. According to the results of a study amongst 731 trainees from different Greek hospitals, residents were generally dissatisfied with the educational environment, while the expectations they had when they entered medical school were not covered [24].

Our study provided empirical support for the existence of stress among residents. Stress symptoms frequency was between moderate and high levels in all SSS subscales, except unfavorable relationships with colleagues which were moderate. As far as the symptoms intensity, this was in medium levels. This complies with other researchers’ findings in Greece. A study on occupational stress amongst 355 Greek trainee doctors showed that they expressed significantly higher levels of stress than other professionals [4]. Findings from other countries’ studies confirm occupational stress among residents [5, 25, 26].

Analysis revealed statistically significant associations between almost all PHEEM and stress dimensions. In all cases, higher frequency and intensity of stress symptoms was positively associated with the negative clinical environment evaluation. The fact that stress is typical during the residency period is well-documented in the literature [27].

The findings indicate a significant positive correlation between total SC and Perceptions of autonomy and social support dimensions of PHEEM. Additionally, Participation in the Community was positively related with all three PHEEM dimensions, and “Tolerance to Diversity” to perceptions of autonomy subscale. The absence of similar studies makes the comparison with previous findings impossible. However, results are in line with SC theory basic assumptions. Higher SC levels may influence individuals’ tendency for autonomy and providing and taking social support, and therefore their perceptions of autonomy and supportiveness opportunities [28, 29]. For example, an active actor in the local community maintains this tendency is the working environment, which in turn may lead to greater opportunity to reach higher levels of autonomy and social support.

In summary, findings suggest that medical residents perceptions concerning their educational environment are negatively associated with almost all stress subscales. Preliminary evidence suggests that higher levels of SC are associated with the positive evaluation of their educational experience. Further research, regarding both individuals’ and organizations’ SC on trainees perceptions, is suggested.

## Limitations

- The study was cross-sectional, so the results cannot be generalized.
- Participants were selected on the basis of convenience.
- The sample size was relatively small.
- Only individual SC was measured. It would be helpful to be treated both as an organizational resource (SC of the hospital).

## Abbreviations

SCQ: Social Capital Questionnaire
PHEEM: Postgraduate Hospital Educational Environment Measure
SSS: Sources of Stress Scale
SC: social capital
